# The Criteria People Use in Relevance Decisions on Health Information: An Analysis of User Eye Movements When Browsing a Health Discussion Forum

**DOI:** 10.2196/jmir.5513

**Published:** 2016-06-20

**Authors:** Wenjing Pian, Christopher SG Khoo, Yun-Ke Chang

**Affiliations:** Wee Kim Wee School of Communication and Information Nanyang Technological University Singapore

**Keywords:** information seeking behavior, media, social, Internet, judgment, decision-making, criteria, relevance assessment, consumer health

## Abstract

**Background:**

People are increasingly accessing health-related social media sites, such as health discussion forums, to post and read user-generated health information. It is important to know what criteria people use when deciding the relevance of information found on health social media websites, in different situations.

**Objective:**

The study attempted to identify the relevance criteria that people use when browsing a health discussion forum, in 3 types of use contexts: when seeking information for their own health issue, when seeking for other people’s health issue, and when browsing without a particular health issue in mind.

**Methods:**

A total of 58 study participants were self-assigned to 1 of the 3 use contexts or information needs and were asked to browse a health discussion forum, HealthBoards.com. In the analysis, browsing a discussion forum was divided into 2 stages: scanning a set of post surrogates (mainly post titles) in the summary result screen and reading a detailed post content (including comments by other users). An eye tracker system was used to capture participants’ eye movement behavior and the text they skim over and focus (ie, fixate) on during browsing. By analyzing the text that people’s eyes fixated on, the types of health information used in the relevance judgment were determined. Post-experiment interviews elicited participants’ comments on the relevance of the information and criteria used.

**Results:**

It was found that participants seeking health information for their own health issue focused significantly more on the poster’s symptoms, personal history of the disease, and description of the disease (*P*=.01, .001, and .02). Participants seeking for other people’s health issue focused significantly more on cause of disease, disease terminology, and description of treatments and procedures (*P*=.01, .01, and .02). In contrast, participants browsing with no particular issue in mind focused significantly more on general health topics, hot topics, and rare health issues (*P*=.01, .01, and .01).

**Conclusion:**

Users browsing for their own health issues used mainly case-based relevance criteria to relate the poster's health situation to their own. Participants seeking for others’ issues used mostly general knowledge–based criteria, whereas users with no particular issue in mind used general interest– and curiosity-based criteria.

## Introduction

People are increasingly seeking for and accessing health information on the Internet. A telephone survey of US adults conducted in 2012 by the Pew Research Center Internet & American Life Project [1] reported that 1778/3014 (59%) of US adults had sought health information on the Internet in the past year. The Pew survey found that among the people who looked for Web-based health information, 942/1778 (53%) had sought information about specific diseases or problems, 764/1778 (43%) for particular medical treatments, 480/1778 (27%) for weight loss and control information, 338/1778 (19%) for food safety information, and 268/1778 (15%) for drug information. However, it is not clear how people decide what health information is relevant for particular purposes (eg, diagnosis) and in different situations and what relevance criteria they use.

Relevance, relevance judgment, and relevance criterion are difficult concepts to define. It was thought as a vague but well-understood term without a clear definition. In other words, researchers have difficulty defining the term, but users have no trouble deciding whether a document or piece of information is relevant. For the purpose of this study, we define relevance as a user’s subjective assessment of the relation between a piece of information or document and the user’s situation. A relevant piece of information has a positive relationship to the user’s situation, in that it evokes a positive sentiment in the user. In this study, relevance is operationalized in 2 ways:

1. Evaluative relevance: a piece of information or document is deemed relevant if the user says it is relevant.

2. Predictive relevance: a document is deemed likely to be relevant or to contain relevant information if the user selects or clicks on a document surrogate (eg, document title) to view the full document content.

We define a relevance criterion as a reason that contributes to the user’s relevance judgment. However, the reason can be expressed at different levels of abstraction. High-level reasons include usefulness, topicality, and quality. This study examined the detailed information in the text as information cues that affect relevance judgment. Hence, in the context of this study, relevance criteria are the pieces of information (expressed in the document) that the reader uses to decide whether the information or document is relevant.

Most of the previous studies on relevance judgment were in the context of information retrieval in Web-based bibliographic and full-text databases [2,3] and more recently in Web-based search engines [4,5]. With the rising popularity of social media websites, it is important to investigate the relevance criteria that people use when browsing social media websites.

There are different types of social media applications with different functional characteristics, resulting in different user behaviors. This study focused on a health discussion forum—HealthBoards [6]. The user posts on discussion forums are organized by topics and subtopics in a hierarchical structure. Users of a discussion forum focus on browsing by topic the posts and responses to posts, rather than checking only the responses to their own posts or the posts of specific people, as in Facebook.

Studies on health information seeking have found that people sometimes purposefully seek health information for their own health issues [7] and at other times encounter useful or interesting information serendipitously [8]. People were found to seek health information in the Internet sometimes for themselves and sometimes for others [1]. This study distinguished between these 3 types of health information seeking context or needs: (1) seeking information for the user’s own health issue, (2) seeking information on behalf of other people (ie, for someone else’s health issue), and (3) browsing with no particular health issue in mind. Although systematic review for Web-based health community users suggests that there might be different participation styles among users for different topics [9], this user context or information needs dimension is applicable to any health topic.

Thus, the objective of this study was to find out the relevance criteria that people use to make relevance judgments on a health discussion forum, in these 3 types of use context.

The results of this study carry implications for the design of more user-oriented health information systems. As it was found that people with different types of use contexts focus on different types of health information, the relevance ranking by the search function can assign different weights to different types of information, depending on whether the user is searching for self, others, or with no particular issue in mind.

### Prior Work

This section reviews 3 areas of research: studies of relevance criteria, studies of information behavior using eye tracker systems, and factors influencing the Web-based health information behavior.

#### Studies of Relevance Criteria

Relevance criteria are factors that contribute to users’ relevance judgments [10]. They can also be thought of as the clues that people look for to infer relevance [11]. Researchers have investigated users’ subjective relevance judgments in various kinds of settings (eg, students, working people, and different occupations) [2,3]. On the basis of their studies, several lists of relevance criteria have been derived.

A list of 21 categories of relevance criteria were derived from an interview of 30 people who used weather information in their jobs about how they judged relevance of information from various kinds of media, such as TV and newspaper. They can be grouped into 10 groups: accuracy, currency, specificity, geographical proximity, reliability, accessibility, verifiability, clarity, dynamism, and presentational quality [3].

Seven groups of evaluation criteria were identified from an interview of 18 students and faculty members to identify how they judged document relevance in preparing assignments [4]: information content, user’s previous experience and background, user’s belief and preference, other information and sources in the document, the source of the document, the document as physical entity, and the user’s situation. Several user aspects were identified in the study, which indicate that the users’ own characteristics can influence their relevance judgment, not just the objective features of the information itself.

Further studies were carried out to find out how students made relevance assessments. History-major graduate students were recruited, and they made 27,000 relevance assessments on interview segments of Holocaust survivors and real user topics [12]. Four types of relevance were identified: direct relevance, indirect relevance, context relevance, and comparison relevance. It was noted that comparison relevance makes use of similarity criteria to help users understand the topic. They divided comparison relevance into external factors (such as time and place), the factors of participants, and factors of act and experiences (such as attitude, feeling, treatment, and experiences of activities). Comparison relevance may be used by users of a health discussion forum to assess the similarity of the poster’s health condition (based on the description in the post content) to the user’s own condition. In addition, the user’s current treatment regime and experiences may be important criteria in his or her relevance judgment.

Other studies have found relevance criteria that are related to the work environment, task, problem situation, and emotional state of the user. One study was conducted to investigate how users judge information relevance in the social Q&A website, Yahoo! Answers [5]. It identified 6 groups of criteria by analyzing the content and attributes of the questions and answers: content, cognitive, utility, information sources, extrinsic and socioemotional, most of which have been identified in previous studies. However, it was found that the socioemotional group included the social aspects of the environment, and the importance of each relevance criterion varied depending on the topic and environment.

These relevance criteria belong to rather broad high-level categories. They can have different meanings in different contexts. No specific health information relevance criteria were proposed in previous studies. This study has derived a list of detailed relevance criteria used in assessing user-contributed health information (the details of the derivation, and the list is shown in Appendix 1).

#### Information Behavior Research Using Eye Trackers

In the information behavior research area, there have been a few studies that investigated users’ eye movements on information objects to understand users’ mental processes such as relevance judgment and quality judgment. The duration of eye fixation has often been used as an indicator of user’s mental information processing in relevance judgment.

A few researchers have carried out content analyses of information focused on by users [4,13]. Eye movement data were combined with users’ post-experiment commentary on their feelings, thoughts, and intentions when performing particular actions on the screen [13]. It was found that eye fixation was associated with cognitive processing, and that a replay of the eye movement recording helped participants to recall the instances of information encountering. In this study, we assumed that when people’s eyes focus on a particular text passage, they are interpreting the information conveyed by the passage and making use of it in the relevance decision-making process.

A method was developed to connect eye movement data to users’ relevance criteria used during the judgment process when a study was carried out to find out how people make relevance judgments on search results from the search engine, Google [4]. The study participants were asked to think aloud during their search session. The surrogate records in the search result screen with eye fixations were coded by duration and number of fixations and associated with relevance criteria identified in the verbal protocol. They found that eye movements, particularly the attributes of eye fixations (number of fixations and their duration and frequency) reflected users’ relevance dynamics. For example, users put less effort on information related with topicality when they examined the search results, as the surrogate records displayed were assumed to be on the topic. However, when deciding that a surrogate record is not relevant, users tended to have longer fixation duration when considering topicality and scope.

This approach of linking qualitative data of users’ verbalization with the analysis of eye movements can yield more insights into the relevance decision process. However, when people think aloud, they tend to think differently than in natural situations [14]. People were found to have different reasoning processes than the normal reasoning process when they were required to think aloud. Hence, the think aloud process was replaced in this study with a post-experiment user commentary on what they were thinking during the browsing process.

#### Factors Influencing the Web-Based Health Information Behavior

Previous studies found that several kinds of factors influence different aspects of consumers’ health information behavior. Gender was found to influence the frequency of use of the Internet for health information [15,16]. Women were found to have more health information–seeking behaviors than men. Cultural difference was found as a factor influencing what kind of health information platform people prefer to use [17]. Eastern culture was found to show preference to user-contributed health information such as health social media websites, whereas western culture was found to show preference to expertise-based platforms such as WebMD. Familiarity with the topic was found to influence the search efficacy. If more familiar with the health topic, less modification was made during health information–seeking process [18]. However, there was no study on the particular factor—different types of use context or information needs mentioned previously. This factor exists in most Web-based health information–seeking behavior. Once you start seeking health information in the Internet, you search for your own problem, other’s problem, or without a particular problem and for fun. It is important to know how this factor influences people’s judgment on the relevance of Web-based health information—a particular kind of health information behavior.

## Methods

### Framework

This is a study of consumer health information seeking on a particular type of social media website—a health discussion forum. This study focused on the relevance criteria used by people when browsing for health information. People’s eye movement behavior of skimming over the text and focusing on particular text passages during browsing was captured with an eye tracker machine. Content analysis of the data was performed to identify the kinds of information people skimmed over and focused on when making relevance judgments, that is, deciding whether a post contains relevant information to infer the relevance criteria used during the process.

The structure of a discussion forum bears some similarity to information retrieval systems. The user can select a topic by either entering a query keyword in a search box or browsing a classified directory of topics and subtopics. Having selected a topic, the system displays a summary result screen displaying a list of post surrogates (usually the post header, including the title, author, and the number of user views and replies). The user has to scan the post surrogates to select posts that are likely to contain relevant information. When a post surrogate is clicked on, the system displays a detailed post screen showing the content of the main post and response posts from other users. This is sometimes called a discussion thread. In HealthBoards.com, the responses to a post are appended to the end of the post content page, so a post and its responses are equivalent to a full-text document in an information retrieval system. The user reads the post and its responses to identify relevant information. The user also consumes the information by learning something.

The framework used in this study to distinguish between the different stages of health information seeking within a health discussion forum and the 2 types of relevance judgments associated with each stage are summarized in Table 1.

**Table 1:** Stages of health information seeking within a health discussion forum and the 2 types of relevance judgment associated with each stage.

Stage	User information behavior	Relevance judgment
**Stage 1. Searching or browsing**		
	Searching or browsing	System retrieves and displays document or post surrogates matching the search query or selected directory category (relevance judgment by machine)
**Stage 2. Scanning (the document or post surrogates in the summary result screen)**		
	Skimming (over the list of surrogates)	Select surrogates for attention or focus (unconscious relevance judgment based on keywords in the text that catches the eye)
	Examining (individual surrogates)	Select surrogates to retrieve the associated (linked) document (predictive relevance judgment based on an estimation of the likelihood that the document contains relevant information)
**Stage 3. Reading (the document or post content)**		
	Skimming (through the document or post content quickly)	Select text for attention or focus (unconscious relevance judgment)
	Examining (and absorbing the information in the document or post content)	Deciding whether the information is relevant or likely to be useful in a use context (evaluative relevance judgment)

This framework of user searching and browsing in an information system was derived from 2 frameworks: the 2-stage relevance judgment model [19,20] and the 2-stage browsing model [21,22].

A 2-stage relevance judgment model was proposed for Web-based information searching, based on previous work [23,24]. In this model, the users’ overall relevance judgment of a document retrieved by an information system is divided into 2 stages, predictive relevance judgment and evaluative relevance judgment, as detailed in the following section:

*Predictive relevance judgment* of the document surrogate: the user scans the document surrogates (mainly document titles) retrieved by the information system to make judgments of the likelihood of relevance of the content of the documents. On the basis of the estimated likelihood, the user may retrieve the full text of selected documents to read.

*Evaluative relevance judgment* of the document content: the user reads some or all the text in the document and makes a relevance judgment based on the information in the text.

These 2 stages reflect the typical design of information retrieval systems that require the user to first assess the list of document surrogates in the summary search result screen for potential relevance and then select (ie, click on) document surrogates that are potentially relevant to read the detailed document content. Predictive relevance judgment is based on an estimation of the likelihood of relevance. This kind of estimation is based on limited information provided by the surrogates, which do not provide enough information for full relevance judgment. On the other hand, evaluative relevance judgment is based on full information in the document. The different nature of these 2 types of relevance judgment may involve different relevance criteria or different types of reasoning.

This 2-stage relevance judgment model is suitable for the study of relevance judgments in health discussion forums because the forum systems typically offer 2 kinds of pages: the summary result page, which lists post surrogates (with post title, number of user replies, number of views, and time of posting) and the detailed post page with full content of a particular post and replies from other users.

The second part of our research framework recognizes 2 types of eye movements—skimming and examining—during information browsing. This skimming or reading model was developed to investigate users’ relevance judgments during the information seeking process [21]. It categorized people’s reading of retrieved documents into several types of skimming and reading behaviors, according to whether the user’s eye gaze moves quickly or is statically focused. It calculated the ratio of cumulative reading to cumulative skimming as a measure to predict the user’s relevance decision on a particular document. They found that the ratio of examining to skimming is positively associated with the likelihood of judging information as relevant. This study makes use of the term *examining* instead of the term *reading* used in their study to better reflect that the user is closely focused on particular pieces of text information in contrast to skimming. We use the term *reading* for the entire stage of checking the detailed post content.

Skimming and examining are found not only in the reading stage (ie, reading the document content) but also in the stage of scanning search surrogates. Students were found to exhibit skimming and examining during not only the stage of reading the retrieved full-text documents but also the stage of checking document surrogates to estimate likelihood of relevance [22].

In this study, the 2 types of eye movement behavior, skimming and examining, were incorporated into the 2-stage relevance judgment model, as summarized in Table 1.

### Study Setting

This study chose a particular health discussion forum—HealthBoards [6] as the representative. It was chosen from 10 candidates of health forums searched by Google search engine. The details of the 10 candidates are shown in Appendix 2. The HealthBoards.com was chosen for its huge number of users, frequency of use, comprehensiveness, and ranking on rating websites. The details of these reasons are detailed in the following points:

Number of registered users: 1,079,219 registered users as of September 20, 2015. The second largest was PatientsLikeMe with 300,000 registered users.
Number of posts: 879,065 threads, and 4,874,692 posts and replies.
Number of subsections on particular health conditions and problems: more than 280 subsections.
Number of daily Internet users: 3000+.
Ranking: No. 1 health forum in Yahoo Health search.

This study recruited research participants from residents who were living in Singapore. Requirements were set to carry out the study successfully:

Age: 18 to 50 years;
Education level: either undergraduates or graduates;
Health condition: no critical disease, including HIV, cancer, and so forth;
English fluency: competent in English reading and speaking.

The health condition criterion was used to ensure that the participants did not have a debilitating or critical disease because such patients are expected to have rather different needs and information behavior. The English fluency was used to ensure the participants understand clearly the English text on this health forum.

### Study Design

The steps of this study were adapted from previous studies using eye tracker machines [13,21] as follows:

Brief the participant on the general purpose of the study.

Ask the participant whether he or she has some health issue to seek information for in the discussion forum. If no, ask whether the participant wants to seek information for the health issue of a relative or friend. If the participant cannot think of a health issue for self or others, then, ask the participant to browse the discussion forum for health information. Ask the participant to describe the health issue in some detail if the participant is seeking for own health issue or other’s health issue.

Give the participant an introduction to the eye tracker machine—what it does, how it works, and what the participant should do to obtain accurate results.

Calibrate the eye tracker machine for the participant, following instructions in the eye tracker manual. The calibration is used to adjust the eye tracker system to the participant’s gaze positions. The system indicates whether the calibration is successful.

Ask the participant to look for information in the discussion forum that is relevant (or browse for interesting health-related information).

This study did not set a time limit for participants. They kept browsing until they felt satisfied with the information they found or decided to end the session. The average duration of a session is 9.5 minutes (ranging from 4.1 to 14.6 minutes).

Replay a video recording of the participant’s search and browse session together with the indications of eye gaze positions and eye movements. Ask the participant to comment on the post surrogates with eye fixation as well as those selected and the text passages in the post content with eye fixation. The purpose was to identify the participant’s reasons why a post surrogate or detailed post is relevant or not. More details of the questions are summarized in Table 2.

**Table 2:** Interview guide to obtain participants’ comments on the video recording of his or her eye movement behavior.

Screen	Questions to ask
**Summary result screen of post surrogates**	
	For post surrogates with eye fixation, ask *What attracted your eyes to these posts? Why is this interesting?*
	For post surrogates clicked on, ask *Why did you select this post for further reading?*
	Look through the post surrogates without eye fixations and point to a few titles that appear similar in topic to the post surrogates the participant had clicked on, and ask *Why was this post not interesting?*
**Detailed post screen**	
	Point out text segments in the post content with eye fixation and ask *Is this information useful or relevant?*
	Finally, ask *Why do you think this post is relevant or not relevant?*

### Study Population

This study was targeted at laymen who did not have severe or critical diseases. A layman refers to normal adults who were not health professionals or did not hold expertise in health-related areas. Their behaviors were thought of as consumer health information behaviors.

They are thought not to have severe or critical diseases because most of the times people who had these diseases would directly refer to the doctors rather than browsing the Internet for related health information. Besides, they are well educated because the text in health forums may require some level of language comprehension that less-educated people are not equipped with.

### Sampling Technique

This study took convenient sampling method to recruit research participants. The researchers sent out invitation emails to the students and staffs in Nanyang Technological University and made posters on school canteens and libraries. They also invited their friends by telephone calls and messages and asked them to forward the invitation to their friends. Because it will take about 1 hour to finish the experiment with briefing and post-experiment interview (not including the time for transportation), most of the participants are university students as they have more free time, and it takes just a few minutes’ walk in campus for them to get the experiment laboratory.

### Sample Size

Overall, 60 study participants were recruited from students and staff of Nanyang Technological University (a large government-funded university in Singapore). An additional 10 participants were recruited from our network of friends who were working adults outside the university. The participants were recruited by advertisements posted on the university campus notice boards and by email, telephone, and oral invitation. The 60 study participants comprised 32 full-time students, 14 part-time students, 4 staff members, and 10 working adults from outside the university.

Two participants failed in the calibration of the eye tracker machine and were excluded from the study, leaving 58 participants who contributed data to the study.

### Ethical Consideration

An approval from Institutional Review Board of Nanyang Technological University was obtained before the formal study was carried out as user’s eye movements and their feedbacks and comments were used in this study. Besides, they were asked to provide the information about their own problem or their friends and relatives. Hence, an informed consent was asked to sign after the briefing was finished. If they decided not to sign, they would leave this study with no personal information recorded.

### Data Analysis and Management

Content analysis of the kind of texts that the participants focused was carried out to find out what kinds of health information they paid attention to and possibly use in the relevance judgment process. In the content analysis, the sentences that participants fixated on were extracted from the screenshots and categorized into different types of health information by 2 coders.

**Figure 1:**
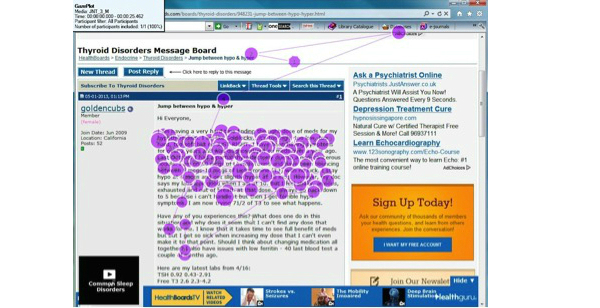
Screenshot of detailed post page with participant’s eye fixations.

The eye tracker machine recorded participants’ eye movements on each screen and generated a static image of each screen with round spots indicating eye fixations and lines indicating quick eye movements, as illustrated in Figure 1. The size of the round spots reflects the relative duration of the eye fixation. Post surrogates and text passages in the detailed posts covered with round spots were considered to be examined and interpreted by the participant and used in the participant’s relevance decision. Surrogates and text passages included in the lines of quick eye movements were considered to be skimmed by the participant.

Different types of health information are related to different aspects of health issues, such as symptoms and history of disease related to the user’s condition, drug names, and treatments, which can be considered factual knowledge, and diet- and exercise-related information, which are general interest or lifestyle topics. Content analysis of users’ commentary provides more support and explanation for the results of the eye movement analysis. We had derived a comprehensive coding scheme in the pilot study to be used in the content analysis of participants’ eye movements. The details of derivation are included in Appendix 1 [1,25-27].

#### Analysis of Post Surrogates and Post Content With and Without Eye Fixation

The data were downloaded from the eye tracker machine and coded into different types of health information.

Because each participant fixated on and skimmed over different total number of post surrogates and detailed posts, we could not directly use the count of post surrogates and detailed posts containing a particular type of health information for comparison. Instead, we calculated the average percentage of each type of health information found in the post surrogates and the detailed posts for each group of participants. This was computed in 2 steps:

1. Compute the percentage of each type of health information for each participant within a group of participants: count the post surrogates that were fixated on containing a particular type of health information and divide by the overall count of all post surrogates that were fixated on.

2. Compute the average percentage for each group.

The purpose was to find out whether different types of health information were used in the different use contexts and the importance of particular types of health information (ie, relevance criteria) in making relevance judgments.

A screenshot of a detailed post page showing text with eye fixations is shown in Figure 1. The coding was conducted by 2 coders. To check intercoder reliability, both the coders were asked to code about 20% of the data (the data for the first 12 participants). Cohan’s kappa between the 2 coders was found to be .82, indicating good consistency. The conflicting codings were discussed with the coders to help them get a clearer conception of the categories. For the purpose of computation, we resolved the conflicting categories by selecting the category after reviewing with the 2 coders. The remaining 80% of the coding data were divided evenly between the 2 coders.

#### Analysis of Participant Comments From the Post-Experiment Interview

The participants’ comments on whether and why particular post surrogates or post content was relevant were analyzed. The purpose was to elicit the criteria and reasons for their relevance judgments and find out what they thought about the health information on the screen during browsing.

## Results

The results of the content analysis indicate that the 3 groups of participants focused on different types of health information: participants seeking for their own health issue focused mostly on symptoms, history of disease, and treatment, which can be considered case-based relevance criteria that participants might use to match their own conditions. Participants seeking for others’ health issue focused mostly on treatments, medical terms, and cause of disease, which could be considered general knowledge–based relevance criteria. Finally, participants with no particular health issue in mind focused mostly on general health topics such as diet and exercise, public awareness topics such as smoking and air pollution, and interesting stories. These could be considered general interest–based relevance criteria.

Before the detailed quantitative results of this study, the demographic information of the 58 participants is summarized in Table 3. Most of the participants were Singaporean Chinese (Singapore citizens) and Chinese nationals. The Singaporean participants were all educated in English-medium schools. Chinese nationals were educated in Mandarin in their elementary school in China but took English courses from secondary school to college. Before they came to Singapore, they had to pass 1 or more English tests such as International English Language Testing System and Test of English as a Foreign Language.

**Table 3:** Summary of demographic information of research participants (N=58).

Characteristic	Demographics	N
**Nationality**		
	Chinese	31
	Singaporean	24
	Others	4
**Degree**		
	Undergraduate	15
	Master degree	28
	PhD	15
**Occupation**		
	Full-time student	30
	Part-time student	14
	Staff	4
	Working adults	10
**Age, years**		
	18-20	2
	20-30	27
	30-40	25
	40-50	4

### Scanning Post Surrogate Page

**Figure 2:**
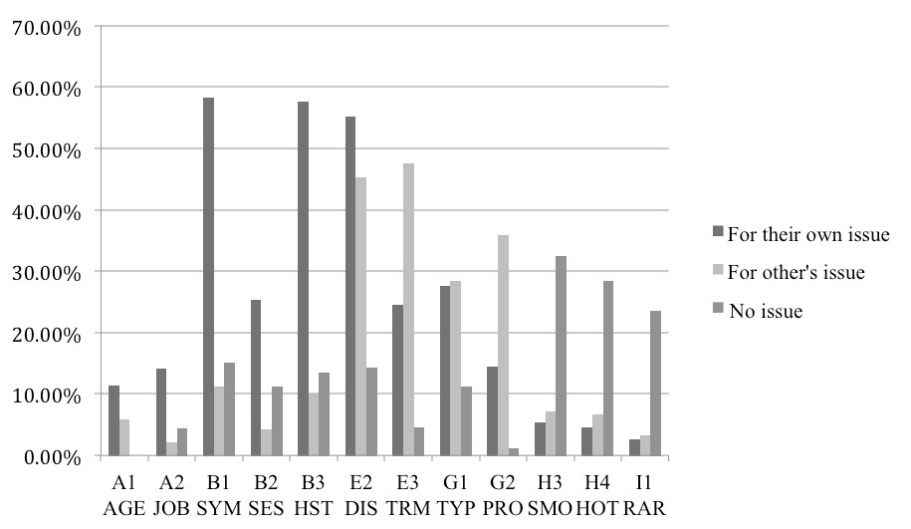
Percentage of post surrogates containing each type of health information for the 3 groups of participants.

The percentages of post surrogates containing each type of health information in the scanning stage are shown in Figure 2. Categories with percentages less than 5% were removed from the graph so that it can fit in the page.

It can be seen in Figure 2 that the 3 groups of participants with different types of use contexts focused on different types of health information. For participants seeking for their own health issue, the following were the most common categories they fixated on (the 95% confidence intervals [CIs] are summarized in Table 4):

Description of patient symptom (B1 SYM: 58.3%).
Personal history of disease (B3 HST: 57.6%).
Description of disease (E2 DIS: 55.2%).

In comparison, for the participants seeking for other’s health issue, the most common types of information fixated on were as follows:

Description of disease (E2 DIS: 45.3%)
Description of terms (E3 TRM: 47.5%)
Description of procedure used (G2 PRO: 35.8%).

For participants browsing with no particular issue, the most common types of information fixated on were as follows:

Smoking issue (H3 SMO: 32.4%)
Hot health topic (H4 HOT: 28.4%; eg, sexual issue, weight control, exercise, diet)
Rare health issue (I1 RAR: 23.5%).

A few other types of health information were also found important for each group of participants (with frequency above 20%):

Participants seeking for their own health issue: subjective feeling of having the disease, description of treatment, and disease terminology.
Participants seeking for other’s health issue: type of procedure.

There was a possibility that the percentages for the different types of health information with eye fixation merely reflected the overall distribution in the discussion forum. The *t* test was carried out to compare, for each type of information, the percentage of the surrogates skimmed versus the percentage of the surrogates fixated on. As summarized in Table 4 , the types of health information with higher percentage in the set of surrogates fixated on had significantly lower percentage in the set of surrogates skimmed (eg, SYS symptom 29.3% with a 95% CI of 28.5-29.7 in skimmed surrogates vs 58.3% with a 95% CI of 58.1-58.6 in fixated surrogates). The other types of health information summarized in Table 4 hold the similar patterns. This indicated that the participants with different types of use context intentionally focused on the particular types of health information in making relevance judgments. The other types of health information were not found to be significantly different in fixated and skimmed post surrogates.

**Table 4:** Percentage of health information in the set of skimmed surrogates compared with the fixated surrogates.

Reason	Category of health information	% In surrogates skimmed (95% CI)	% In surrogates fixated on (95% CI)	*P* value
**For their own heath issue (N=18)**				
	B1 SYM symptom	29.3 (28.5-29.7)	58.3 (58.1-58.6)	.01
	B3 HST history of disease	18.4 (17.2-18.6)	57.6 (57.2-58.0)	0
	E2 DIS description of disease	29.1 (28.3-29.9)	55 (54.3-55.7)	.02
**For other’s health issue (N=18)**				
	E3 TRM terms	25.1 (24.1-26.1)	47.5 (47.2-47.8)	.01
	E2 DIS description of disease	17.6 (17.3-17.9)	45.3 (45.0-45.6)	.01
	G1 TRT treatment	12.5 (12.0-13.0)	28.4 (28.2-28.6)	.02
**No particular issue (N=22)**				
	H4 HOT hot topic	16.5 (15.6-17.4)	32.4 (32.0-32.8)	.01
	H3 SMO smoking	17.3 (16.5-18.1)	28.4 (28.0-28.8)	.01
	I1 RAR rare issue	13.2 (12.6-13.8)	23.5 (23.0-24.0)	.01

We further calculated the percentages of different types of health information for the selected post surrogates (ie, clicked on). The selected post surrogates were a subset of the post surrogates with eye fixations. For the selected post surrogates, the most common types of health information found were:

*For participants seeking for own health issue*

Description of patient symptom (B1 SYM: 88%)
Personal history of disease (B3 HST: 75%)
Description of disease (E2 DIS: 72%).

*For those seeking for other’s issue*

Descriptions of terms (E3 TRM: 77%)
Cause of disease (E1 RSN: 72%)
Description of procedure (G2 PRO: 46.7%).

*For those with no particular health issue*

Hot public health topics (H4 HOT: 68%)
Smoking issue (H3 SMO: 56%)
Rare health issues (I1 RAR: 28%).

**Figure 3:**
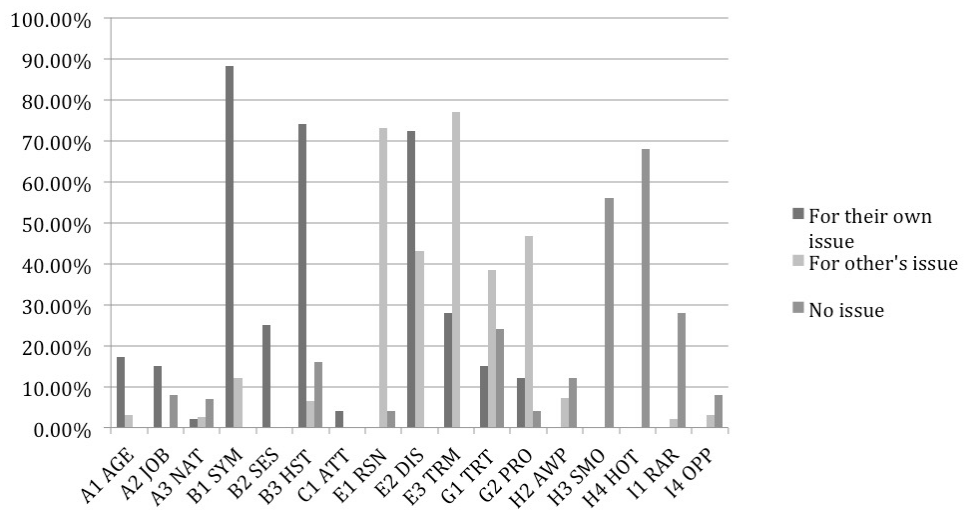
Percentage of selected post surrogates containing each type of health information (the types of health information with less than 5% occurrence are left out).

The most common types of information were mostly the same as those for fixated post surrogates but with even higher percentages. The only difference is in the increased importance of cause of disease for participants seeking for others’ health issue. The details are shown in Figure 3.

For each type of information that had a high percentage in a particular group (eg, description of symptom for participants seeking for their own issue), we calculated the percentage of fixated post surrogates that were actually selected among these 3 groups. If the participant clicked on the post surrogate, it suggested that the participant thought it likely to be relevant. We calculated the ratio of post surrogates selected over post surrogates fixated on by dividing the number of post surrogates containing the type of information that were selected by the number of post surrogates containing the type of information that were fixated on.

For example, if there were 60 surrogates with fixations containing a description of a symptom and of these 30 were selected, the ratio is 50%. The assumption is that if the participant clicked on a post surrogate, the participant thought the post content likely to be relevant.

As a baseline for comparison, we also calculated the overall percentage of fixated surrogates that were selected by dividing the number of post surrogates that were selected by the number of post surrogates that were fixated on.

The results are summarized in Table 5. Analysis of variance was performed to identify significant differences among the 3 groups of participants, and the *P* values obtained are also summarized in Table 5.

It was found that participants seeking for their own health issue use personal history of disease (*P*=.01), description of patient symptom (*P*=.02), and the description of disease (*P*=.17, but *P*_1,2_<.01 and *P*_1,3_=.01) significantly more in determining the relevance of post surrogates. Participants seeking for other’s issue used significantly more the treatment information (*P*=.01), cause of disease (*P*=.03), and disease terminology (*P*=.11, but *P*_1,2_and *P*_2,3_=.01) in the relevance judgments. In contrast, participants browsing without a particular issue used a general-interest topic, smoking (*P*=.01), hot topic (*P*=.01), and rare topic (*P*=.02). Thus, participants with different use contexts used significantly different kinds of relevance criteria in their relevance decisions.

**Table 5:** Percentage of fixated surrogates that were selected for each type of health information.

Type of health information^a^	For own health issue (group 1, N=18), %	For other’s health issue (group 2, N=18), %	No particular issue (group 3, N=22), %	ANOVA^b^ *P*-value (sig.)	Post hoc *P*_1,2_	Post hoc *P*_1,3_	Post hoc *P*_2,3_
B1 SYM symptom	37	11	2	.02	.01	.00	.01
B3 HST history	32	14	4	.01	.01	.00	.00
E2 DIS description	30	14	11	.17	.01	.00	.18
E3 TRM terms	7	29	4	.11	.01	.45	.01
G1 TRT treatment	16	35	4	.01	.01	.01	.00
E1 RSN cause	4	45	22	.03	.01	.02	.01
H3 SMO smoking	2	7	52	.01	.01	.00	.00
H4 HOT hot topic	1	6	64	.01	.01	.00	.00
I1 RAR rare	3	10	24	.02	.01	.01	.00
**Overall percentage**	15	12	8	.37			

^a^Types of health information with low percentage of occurrence in the fixated surrogates are excluded.

^b^ANOVA: analysis of variance.

### Reading Post Content Stage

**Figure 4:**
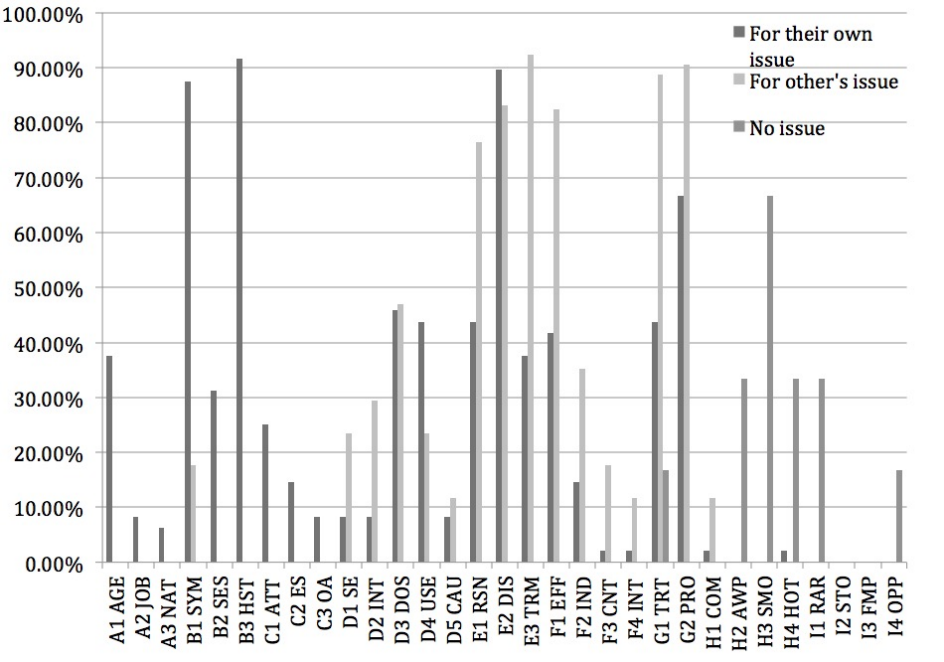
Percentages of particular types of health information in relevant posts.

Content analysis was carried out on the posts that were judged relevant by participants, that is, that the participants indicated were relevant during the post-experiment interview. Figure 4 shows the percentages of the different types of information among all the relevant posts.

From Figure 4 , it can be seen that the situation was similar to that of the scanning stage. For people seeking for their own health issue, the most common types of health information that participants fixated on were personal history of disease (91.7%), description of disease (89.6%), and description of patient symptom (87.5%).

For participants seeking for other’s health issue, the most common types of health information were description of terms (92.3%), description of procedure (91.5%), and description of treatment (88.7%).

For participants with no particular health issue, the most common types were smoking (66.7%), hot topics (33.7%), and air and water pollution (33.7%).

**Figure 5:**
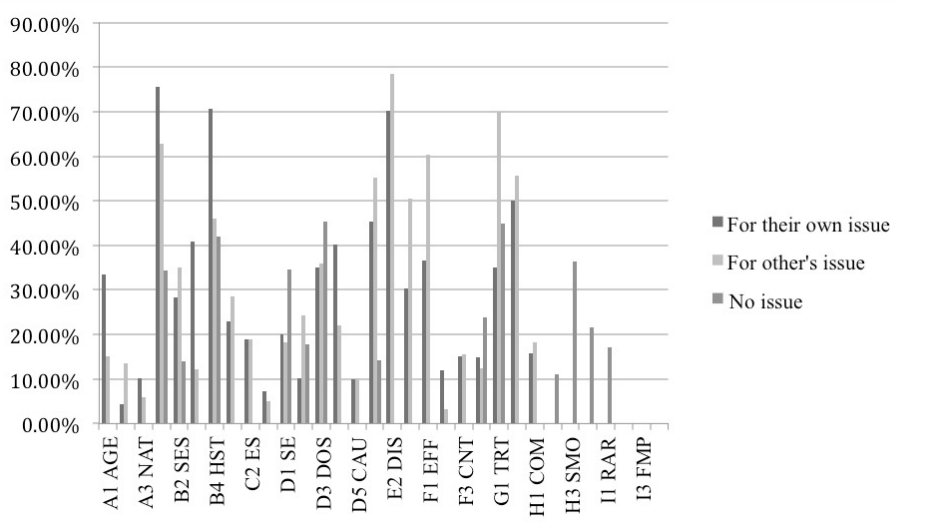
Percentages of posts (read) containing each particular type of health information.

For comparison, the percentages of posts that were read containing each type of health information are shown in Figure 5. It was found that for participants seeking for others’ health issue, the percentages of posts that were read containing personal or case-related types of health information were higher compared with the percentages of posts judged as relevant. In fact, the percentages of posts judged as relevant were nearly zero, which indicated that participants seeking for others’ health issue treated personal details as a sign of nonrelevance. Other than this, the patterns were similar with those for posts judged as relevant.

To further investigate the importance of particular types of health information in determining a detailed post’s relevance, we calculated the ratio of posts judged relevant to posts that were read for each type of information by dividing number of posts read and judged relevant, containing this type of information by number of posts read, containing this type of information.

It was found that description of symptoms, personal history of disease, and description of disease were most important types of information for participants seeking for their own health issue (*P*=.001, .001, and .02) but not for the other 2 groups of participants. For participants seeking for others’ health issue, cause of disease, description of terms, and treatment procedure were the important types of information (E1 *P*=.01; G2 *P* <.01; E3 *P*=.23 but *P*_1,2_=.01 and *P*_2,3_=.02). In comparison, air pollution, smoking issue, and rare cases were the important types of health information for participants with no issue in mind (*P*=.02, .01, and .001). The details are summarized in Table 6. These findings were similar to the findings for the scanning stage, suggesting that participants with different types of use contexts used the same types of health information in detailed post reading stage as in surrogate scanning stage as relevance criteria. However, different groups of participants tended to use different types of relevance criteria.

**Table 6:** Percentages of read posts that were judged as relevant for the most important types of health information.

Type of health information	For their own health issue	For other’s health issue	With no particular issue	ANOVA^a^ *P* value	Post hoc *P*_1,2_	Post hoc *P*_1,3_	Post hoc *P*_2,3_
**B1 SYM symptom**	74.5%	32.2%	11.2%	.001	0	.001	0
**B3 HST history**	72.3%	21.4%	5.6%	.001	0	0	.001
**E2 DIS description**	69.1%	31.6%	7.1%	.02	.01	.01	0
**E1 RSN cause**	16.5%	67.2%	9.4%	.01	.01	0	0
**E3 TRM terms**	6.5%	21.3%	4.5%	.023	.01	.44	.02
**G2 PRO procedure**	14.4%	53.2%	2.5%	.001	0	0	.001
**H2 AWP pollution**	4.5%	15.3%	55.9%	.02	.02	0	0
**H3 SMO smoking**	6.4%	4.5%	45.2%	.21	.67	0	0
**I1 RAR rare issue**	2.1%	7.5%	42.1%	.001	.001	0	0

^a^ANOVA: analysis of variance.

### Post-Interview Analysis

The participants were interviewed after the health information seeking session to obtain self-reported commentary on their relevance judgments. Questions were asked to find out what were the reasons that attracted participants’ attention and the criteria or reasons why they judged particular post surrogate or detailed post as relevant.

The participants’ responses were coded to identify 3 broad types of relevance criteria: case-based criteria, basic or general knowledge–based criteria, and general awareness or curiosity-based criteria. To improve coding consistency, the coding was based on the presence of cue words that reflect the 3 categories. For instance, if a participant’s answer included the following terms or phrases “I compare,” “similar to my situation,” “the similarity between mine and the post,” “familiar with the condition/symptom,” and other similar phrases, then, they were coded as case-based criteria. If the answer contained the phrase “I learned before,” “I knew it before,” “I searched for them in the past,” “I heard,” or other similar phrases, they were coded as basic or general knowledge–based criteria. The details of the coding scheme are summarized in Table 7.

The coding was done by the first author and another PhD student in the same school (not the same person as the coders for the content analysis of text with eye fixation). The coders looked for cue words or cue phrases as summarized in Table 7 and used them to categorize participants into these 3 groups of criteria. If cue words and cue phrases belonging to more than 1 category were found, then, they were simultaneously counted as belonging to all the matching categories. A high Cohen’s kappa of .91 was obtained for the 2 coders. Disagreements between the 2 coders were easily resolved by examining more of the participant’s answers.

Table7. Coding scheme for post-experiment interview data.

**Table:** 

Criterion	Keywords or phrases to look for
**Case-based criteria**	
	Compare, match, comparison, similar, consistent Similar to my problem, situation, or condition, same as mine or my problem, situation, condition, or disease, like my problem, situation, or condition
**Basic or general knowledge–based criteria**	
	Know, learn, familiar Heard or learn from, read this before from newspaper, book, magazine, or Internet, somebody told or informed me
**General awareness or curiosity-based criteria**	
	Interesting, funny, rare, weird, strange, curious Smoking, pollution, exercise, diet, fitness Everybody know, hot topic, public concern, environmental, healthy, well-being

Some participants provided detailed explanations of why they judged 1 particular post surrogate or detailed post as relevant. They mentioned in which ways they compared the post with their own conditions, such as age, location of problem, description of the pain, and diagnosis result. However, most of the participants provided only basic reasons. On the basis of the participant’s initial answer, we followed up with more probing questions. As the interview was conducted after the experiment session, the participants were somewhat tired and not many gave very detailed explanations.

From the analysis of the answers, it was found that for participants seeking for their own health issue, 13 of 18 (72%) clearly used condition or symptom match in their judgment of post surrogates and detailed posts. Here is a quote from a participant:

I found the guy in this post was quite similar to me in the position of headache. He also had the problem after a long time of sitting in front of the screen, the same as me. He did some massage but didn’t work for a long time. I also tried this but found not very useful.”

Three participants were ambiguous in their explanation but mentioned some comparison between themselves and others:

When I read the post I realized the boy did not have a history of jogging as me. His pain was due to the injury of his leg. I do not think it is the same reason as me since my pain comes from the muscle.

The remaining 2 participants expressed other reasons for judging relevance. One wanted to find new information he did not know before. Another used the reference from well-known medical journals or websites as the criterion of relevance.

For participants seeking for other’s health issue, 15 of 18 (83%) used medical and health knowledge that they gained from school or previous searching to guide them in the relevance judgment. For example:

I heard from my friend about acid reflux. So I want to know more about this and can share with him what I find here.”

Three of them made the judgment with other reasons. Two mentioned novelty of the information as the reason they judged the post as relevant. The last participant did not trust information from general users of this health discussion forum and wanted to find comments from health professionals.

For participants with no particular issue, 16 of 22 (73%) participants was found to judge relevance of post surrogates and detailed posts by their interest, curiosity, rarity, and some well-known health issues (eg, diet and exercise). For example:

I found it funny since I did not expect that a girl looking to lose some weight would believe that only magic pills can help her. She did not do any exercise and that is impossible.

The other cluster of 6 of 22 (27%) participants judged relevance by a mixture of case-based matching and basic knowledge criteria. Moreover, it was found that these participants actually had latent health information needs. For some posts, they used case-based criteria similar to the participants seeking for their own health issue. For other posts, they used their prior medical knowledge. We labeled these as “participants with latent health information needs.”

Latent health information needs were detected when these participants were asked the reason for reading particular detailed posts. Because they were grouped as participants with no particular health issue, they were expected to respond with reasons related to personal interest and curiosity. Instead, their answers suggested that they had some health issues related to the topics of these detailed posts or had heard from their friends about a health issue. For example:

When I come across the post, I recall I sprained my left foot three years ago. When I read the post, I found that the guy sprained his foot because of running, the same as me. So, I continued to read and found it relevant. I can make use of his experience to avoid sprain in the future.”

Actually my grandma had diabetes, so I want to know if any useful information in the post. I realized the man in the post used diet control and I heard before. Some reply suggests some herb medicine will help the patient to control the level of blood glucose. I think it useful and will tell my grandma.

Their responses indicated that latent health information needs were activated at some point during the information-seeking session when they came across familiar topics that triggered a memory.

Another possibility for the observed latent health information needs is that the participants were reluctant to share their real health information needs with the researcher at the beginning of the experiment. They became more comfortable later after reading posts of the health issue. However, we did not detect clear evidence of this in the study.

## Discussion

It was found in this study that users browsing a health discussion forum made use of different types of information in relevance judgments and exhibited different eye movement patterns in the 3 types of use contexts—seeking information for self, seeking for others, and browsing with no particular issue in mind. Users browsing for their own health issue were found to use mainly case-based relevance criteria such as symptoms, personal history of disease, and description of disease and personalized treatment in their judgments. Participants seeking for others’ health issue were found to use mainly general knowledge–based criteria such as medical terms, cause of disease, and basic treatments and procedures in their judgments. In contrast, participants seeking with no particular health issue were found to be interested in general health topics, hot topics, and rare health issues.

The personalized treatment refers to the customized treatments that patients received based on their unique conditions, whereas basic treatment refers to the general treatment that can be found in medical books or manuals.

Looking at the results in more detail, participants seeking for own health issue focused mainly on the poster’s symptoms, personal history of disease, and description of disease both when scanning post surrogates and reading detailed post content. These case-based criteria could be considered as more detailed categories of the comparison relevance category identified by Huang and Soergel [12]. They defined comparison relevance as the relevance derived from the similarity between 2 different cases. People who are seeking for their own health issue often compare their own situation (experiences, feelings, symptoms, and history of disease) with the description of the poster’s situation.

Participants seeking for other people’s health issue focused on the terminology, description of the disease, cause of disease, and available treatments. These types of information can be considered to be more detailed relevance criteria within the broader category of content or information in the framework of Cool, Belkin, and Kantor. The category of content or information can be interpreted as the factual knowledge of health issues and treatments. When people seek health information for others’ health issue, they are often not familiar with the details of the patient and have to consider only generic medical information in their relevance judgment.

It cannot be concluded that the relevance criteria identified in this study are the only ones used when browsing a health discussion forum. People also use other relevance criteria implicitly, such as topicality, accuracy, presentation, and authority, but, these cannot be determined just from content analysis of text with eye fixation. People seeking for their own issue and other’s health issue must find the right topic before they can check and read other details. Participants sometimes check the poster’s profile, which suggests that authority is also considered. It is likely that these implicit relevance criteria will become the focus of attention when they are violated. However, no instance of this was encountered in this study. These criteria might also be important considerations when users decide to actually use the information or adopt a recommendation in practice.

The results of this study carry implications for the design of more user-oriented health information systems. The relevance ranking by the search function can assign different weights to different types of information, depending on whether the user is searching for self, others, or with no particular issue in mind. The user can be prompted to select one of these use contexts when accessing the system. For users seeking for their own health issue, the summary result page can display the post surrogates that best match the user’s profile, if available. If the user is browsing with no particular topic in mind, the summary result page can display post surrogates that were clicked on and viewed by the highest number of previous users, indicating topics of general interest. A metadata field for posters to indicate the type of health information included in their post can help the system to filter and display posts that better match the use context and health profile of the user.

### Limitations

This study has some limitations that need further exploration and investigation:

The participants are residents of Singapore (most of them are Singaporeans and Chinese nationals). This study did not include participants from other ethnic groups and nationalities.

The participants did not have a critical health problem at the time of conducting the study. People with severe problems may exhibit different relevance judgment behaviors.

The demographic profile of participants did not cover all segments of the society. In particular, the participants were either undergraduate students or had at least an undergraduate degree.

This study did not consider the influence of human factors, such as personality and attitude to the Internet. Two participants were found to have long examining duration owing to their reading habit developed in childhood.

The health information used in this study was written in English. Content in other languages might influence people’s eye movements and corresponding relevance judgments. For example, Chinese characters are quite different from English text in size and ways of organization.

### Conclusions

This study chose a particular health discussion forum—HealthBoards [6]—as the study platform. It is representative of user-contributed content in health discussion forums, as they are similar in structure and content. Hence, the results of this study are very likely to hold with other Web-based health discussion forums. In addition, the results might also be applicable to other types of social media websites (eg, Facebook groups for various diseases) with lots of user-contributed content. People seeking for their own health issue might look for Facebook or blog pages of people with the most similar condition (ie, identify the most similar person rather than the most similar post), as information on Facebook and in blogs is organized by person rather than topic.

In contrast, the results may not be applicable to authoritative health websites maintained by health care organizations and government agencies (eg, Mayo Clinic and PubMed). People seeking for their own health issue on these websites may have difficulty matching their own condition with the description on the websites. They have to use basic medical terms in searching and making relevance judgments.

## Appendix

Multimedia Appendix 1 The derivation of coding scheme.

Multimedia Appendix 2 Health discussion forum candidates searched through Google search engine.

## Abbreviations

A1 AGE: age and gender
A2 JOB: job and occupation
A3 NAT: nationality
B1 SYM: description of patient symptom
B2 SES: subjective feeling of having a problem
B3 HST: personal history of disease
C1 ATT: attitude to the problem
C2 ES: emotional status of knowing the problem
C3 OA: other’s attitude and support
D1 SE: perceived side effect
D2 INT: interaction with another health problem
D3 DOS: dosage used
D4 USE: description of procedure used
D5 CAU: caution and reminder
E1 RSN: cause of disease
E2 DIS: description of disease
E3 TRM: description of terms
F1 EFF: efficacy
F2 IND: indications
F3 CNT: contraindications
F4 INT: interaction with other drugs
G1 TRT: description of treatment
G2 PRO: description of procedure
H1 COM: common health issue
H2 AWP: air and water pollution
H3 SMO: smoking
H4 HOT: hot topics
I1 RAR: rare health issue
I2 STO: interesting story
I3 FMP: famous person
I4 OPP: counter-intuitive information
