# Supplementary material for: The Criteria People Use in Relevance Decisions on Health Information: An Analysis of User Eye Movements When Browsing a Health Discussion Forum
Source: J Med Internet Res. 2016 Jun 20;18(6):e136. doi: 10.2196/jmir.5513 (PMC4932243; doi:10.2196/jmir.5513)
Supplement: Multimedia Appendix 1 [file jmir_v18i6e136_app1.pdf]

Multimedia Appendix 1

Derivation of the coding scheme

The coding scheme used to code different types of health information was divided into three broad categories:

- Types of *case-based health information*,
- Types of *factual knowledge-based health information*,
- And types of *general knowledge-based health information*.

This scheme was derived partly from the literature, and partly from open coding I carried out in the preliminary study.

In the preliminary study involving 14 participants, the sentences with eye fixations were extracted and used as the basis for open coding. The sentences from seven participants were used as a training set to derive the coding scheme, and sentences from the remaining seven participants were used as a validation set to validate the coding scheme.

The three broad categories of health information as well as some detailed categories were adapted from the literature. These categories were used as a starting point for open coding. Each sentence within the training set was assigned to one detailed category under one of the three broad categories. However, when a sentence did not fit any of the existing categories comfortably, a new category was created under one of the broad categories.

The list of categories derived and the coding for the training set were reviewed by my thesis supervisor. The revised coding scheme was applied to the validation set of sentences, and it was found that all the sentences could be coded into the existing categories. The details of these coding categories are discussed below.

Case-based health information

Case-based health information refers to health information related to the patients' conditions, their own experiences, and their emotions. This broad category of health information was adopted from the literature. Fox and Duggan [1] found that American Internet users searched for the experiences other users with similar health conditions. This broad category was divided into three subcategories: *patient demographics*, *patient symptom*, *emotional support* and *patient experiences of drug and treatment*.

Patient demographics include *age*, *job*, *nationality*, and *race*. These categories come from the literature. Fox and Duggan [1] used age, race, educational level, and marital status in their survey report of online health information searching. Kishimoto and Fukushima [22] used *age*, *education level*, *marriage status*, *gender and location* (residential area) in their online drug information studies. Cotton and Gupta [23] also used *age*, *gender*, *race*, and *marital status* in their studies to

investigate online and offline health information users.
I took *age*, *job* and *nationality* as the basic demographic categories in this study, while putting all other categories not mentioned in the literature in the *others* category. The *others* category comprise mostly infrequent types of information such as *race* and *location*. The anonymity of online discussion forums allow users to share and exchange information without knowing personal details. In the postings, *age* and *country* were sometimes mentioned to give readers a general profile and context for interpreting the shared information.

Patient symptoms include *symptom description*, *subjective feeling of having the symptom (condition)*, and *patient history of disease*. The categories of *symptom description* and *patient history of disease* came from previous literature. Fox and Duggan [1] found that American Internet users searched for specific disease conditions. They reported that users of an online Facebook group for diabetes posted their concerns of negative events to find out if their experience matched with those of other users. There is one category of health information not mentioned in previous literature: subjective feeling of (having) the problem/condition. During the open coding process, I found that some posts contained this category of health information—description of problem or condition based on the writer's opinion. It sometimes starts with "I feel like…" or "I think it should be …". It is different from *symptom description* in that *symptom description* is the direct description of the patient's problem or condition that has been confirmed by a health professional to be a symptom of the disease. The *subjective feeling* of having a symptom is a speculation of the user. Hence, this category was added in the sub-section of *patient symptom*.

Emotion support is another aspect of user related health information. It includes attitude to the problem, emotional status of having/knowing the problem, and other people's attitude and support. These categories all come from previous literature, but were reorganized in this study. Chuang and Yang [24] defined emotional support into the following types: *relationship, physical affection, confidentiality, sympathy, listening, understanding and empathy, encouragement* and *prayers*. However, these types are too detailed to be useful in this study. Moreover, *confidentiality, listening, understanding* and *empathy* can be categorized into other's attitude since they represent other's viewpoints. Hence, I used *other's attitude and support* to represent all these detailed categories. However, these categories did not take into consideration the emotional aspect of the post writer. Hence, in this study, *attitude to the problem*, and *emotional status of having/knowing the problem* were added to represent the poster's own emotions.

Patient experience of drug and treatment were not frequently found in previous literature. In prior studies, drug and treatment information usually referred to formal or authoritative drug and treatment information. In this study, *patient*

experience of drug and treatment focuses on the user's self report of taking the drug and treatment, including self-reported feelings and effect. The self-reported feelings and drug effects may not match the effects found in clinical trials and listed on the drug labels. The category of *patient experience of drug/treatment* includes the following subcategories: *perceived side effect, interaction with another health problem of patient, dosage used, description of used procedure,* and *caution or reminder from patient*.

Factual/general knowledge-based health information

Factual/general knowledge-based health information refers to health information that is based on medical or scientific facts, or proven and well-known factual knowledge. This type of health information comes from medical reports, surveys, medical journals and experiments, and has higher credibility than user self-reported health information. I subdivided it into two sub-categories: *etiology* and *drug information*.

Etiology is subdivided into *the cause of disease* and *the formal/professional description of the disease*. These two categories come from the literature. Hudges and Cohen (2011) found that there are professionally reviewed health information on some websites including formal descriptions of diseases and (formal) symptoms of diseases. They also found that the medical terms sometimes were used in the description of disease and drug effects. Hence, I added *terminology* in this category.

Drug information refers to the formal expression of drug names, effects, side effects, drug interaction, contra-indication, etc. These categories come from prior literature of online drug information research. Kishomoto and Fukushima [22] found that Japanese health information users searched online for drug information covering the topics of side-effect, action mechanism, effect on body, effectiveness, generic drugs, drug interaction, drug dosage, and services of pharmacies. In this study, I reorganized them into the following subcategories: *efficacy, indication, contra-indication, interaction with other drugs*, and *pharmacology*. The *action mechanism* was replaced by *pharmacology*. The drug *dosage* has been included in the category of *patient experiences of drug.*

General-awareness based health information

There is another group of health information that is neither related to user's experience of health problem, nor to medical factual knowledge. I used *general-awareness based health information* to represent this broad category of health information. This is subdivided into: *general health issue*, and *curiosity based health issue*.

General health issue is related to public awareness and concern. During the open coding process, I found that 3 participants who had no particular health issue browsed for topics of diet, weight control, and mental health issue. Fox and Duggan [1] found that many US Internet users searched for health information about health and fitness. Hence, I used this term to represent these types of health information. It is subdivided into *common health issue, pollution, smoking,* and *hot topic of general interest*. A common health issue is not really a particular health condition, but a health area that the public is interested in, such as weight control and diet control. *Pollution* and *smoking* are well-known health-related issues. *Hot topic of general interest* refers to health issues that are prevalent or of major social concern, and not limited to particular diseases or topics publicized in the mass media. It includes drug safety and mental disorders such as depression.

Besides general health issues of public interest, I added *curiosity-based health information* in this category, subdivided into: *rare issue, interesting story, famous people* and *counter-intuitive information*.

The details of the coding scheme divided into three broad categories of health information are listed in Table 1.

Table 1. Coding scheme for different types of health information

User case-based relevance criteria

A. Patient demographic

A1. Age & gender

A2. Job & occupation

A3. Nationality

A4, Other categories

B. Patient's symptom

B1. Description of patient symptom

B2. Subjective feeling of having a problem

B3. Personal history of disease

C. Emotional support

C1. Attitude to the problem

C2. Emotional status of knowing the problem

C3. Other's attitude & support

D. Patient's experience of drug/treatment

D1. Perceived side effect

D2. Interaction with another health problem (experienced by patient)

D3. Dosage used

D4. Description of procedure used

D5. Caution or reminder (based on patient's experience)

Factual/general knowledge-based criteria

<u>E. Etiology</u>
E1. Cause of disease
E2. Description of disease
E3. Description of terms

<u>F. Drug information</u>
F1. Efficacy
F2. Indications
F3. Contra-indications
F4. Interaction with other drugs
F5. Pharmacology

<u>G. Treatment information</u>
G1. Description of treatment
G2. Description of procedure

General awareness-based criteria

<u>H. General health issue</u>
H1. Common health issue
H2. Pollution
H3. Smoking
H4. Hot topic of general interest

<u>I. Curiosity-based criterion</u>
I1. Rare issue
I2. Interesting story
I3. Famous people
I4. Counter-intuitive information
