# Supplementary material for: The Criteria People Use in Relevance Decisions on Health Information: An Analysis of User Eye Movements When Browsing a Health Discussion Forum
Source: J Med Internet Res. 2016 Jun 20;18(6):e136. doi: 10.2196/jmir.5513 (PMC4932243; doi:10.2196/jmir.5513)
Supplement: Multimedia Appendix 2 [file jmir_v18i6e136_app2.pdf]

Appendix 2: Health discussion forum candidates searched by Google search engine

10 health discussion forums retrieved using Google search engine	
1.	Discussion forums on the Patient website (http://www.patient.co.uk/forums)
2.	Health Forum (http://www.healthforum.com/)
3.	HealthBoards.com (http://www.healthboards.com/boards/index.php)
4.	eHealth forum (http://ehealthforum.com/health/health_forums.html)
5.	netdoctor (http://www.netdoctor.co.uk/interactive/discussion/index.php)
6.	Consumers of Health Forum of Australia (https://www.chf.org.au/)
7.	Mental Health Forum (http://www.mentalhealthforum.net/forum/)
8.	PatientsLikeMe (http://www.patientslikeme.com/)
9.	Health Informatics Forum (http://www.healthinformaticsforum.com/forum/topics)
10.	Discuss Health Forums (http://www.discusshealth.org/)
